# Psycho-socio-emotional characteristics in high intellectual potential child regarding IQ profile (Homogenous/Heterogenous)

**DOI:** 10.1192/j.eurpsy.2022.1064

**Published:** 2022-09-01

**Authors:** S. Hamdioui, L. Vaivre-Douret

**Affiliations:** 1 Faculty of Society and Humanity, Université de Paris, Department Of Psychology, Paris, France; 2 National Institute of Health and Medical Research (INSERM UMR 1018-CESP), Faculty Of Medicine, Villefuif, France; 3 National Institute of Health and Medical Research (INSERM UMR 1018-CESP), University of Paris-Saclay, UVSQ, Faculty Of Medicine, Villejuif, France; 4 Imagine Institute Necker hospital, Department Of Endocrinology, Paris, France; 5 University Institute of France, (iuf), Paris, France; 6 Faculty of Health, Université de Paris, Department Of Medicine, Paris, France; 7 Necker hospital AP-HP Paris.Centre Université de Paris, Child Psychiatry, Paris, France

**Keywords:** High Intellectual potential, IQ profile, Psycho-socio-emotional, Assessments

## Abstract

**Introduction:**

Few studies have analyzed the psychometric profile (Homogenous/Heterogenous), established by the Wechsler scale in high intellectual potential children (HIP, IQ>130), regarding the psycho-socio-emotional characteristics.

**Objectives:**

We aimed to look at the links between the IQ-profile and the psycho-socio-emotional characteristics in HIP.

**Methods:**

Anamnestic questionnaire and Wechsler-Intelligence-Scale for children (WISC-V) were conducted and analyzed in 58 healthy children with HIP, aged 7-to-13 years-old (mean 10y; SD 1.8). It was possible to distinguished 27 Homogenousvs 30 Heterogenous IQ-profile.

**Results:**

No significant difference between homogenous/heterogonous groups, FIQ was positively significantly correlated with “*Reacting very little emotionally*”, “*Tendency to isolation*”. Visual-Spatial-Index (VSI) with “*Ability to adapt to new people*” (r=-0.4, p=0.02), “*few interests”* (r=0.5, p=0.008). Verbal-Comprehension-Index (VCI) with “*Reacting strongly to frustration*”, “*Difficulties to understand limits*”, “*Separation anxiety*”. A significant difference between homogenous/heterogonous groups was shown regarding “*few interests*” with high rate in the heterogonous group (t= -2.34, p=0.023).

**Conclusions:**

HIP seems to cover specific psycho-socio-emotional characteristics linked to IQ index distribution. Thus, it appears interesting to assess more the emotional and socio-cognitive field to understand these characteristics in HIP children.

**Disclosure:**

No significant relationships.

